# Development and validation of a protocol for optimizing the use of paraffin blocks in molecular epidemiological studies: The example from the HPV-AHEAD study

**DOI:** 10.1371/journal.pone.0184520

**Published:** 2017-10-16

**Authors:** Marisa Mena, Belen Lloveras, Sara Tous, Johannes Bogers, Fausto Maffini, Nitin Gangane, Rekha Vijay Kumar, Thara Somanathan, Eric Lucas, Devasena Anantharaman, Tarik Gheit, Xavier Castellsagué, Michael Pawlita, Silvia de Sanjosé, Laia Alemany, Massimo Tommasino, the HPV-AHEAD study group

**Affiliations:** 1 Cancer Epidemiology Research Program, Catalan Institute of Oncology (ICO)-IDIBELL, L'Hospitalet de Llobregat, Barcelona, Spain; 2 CIBER in primary and secondary prevention of viral induced cancers (CIBERONC), Madrid, Spain; 3 Department of Pathology. Hospital del Mar, Parc de Salut Mar, Barcelona, Spain; 4 Laboratory of cell biology and histology, University of Antwerp, Antwerp, Belgium; 5 Division of Pathology, European Institute of Oncology, Milan, Italy; 6 Mahatma Gandhi Institute of Medical Sciences, Sevagram, Wardha, India; 7 Kidwai Memorial Institute of Oncology, Bangalore, Karnataka, India; 8 Regional Cancer Centre, Thiruvananthapuram, India; 9 International Agency for Research on Cancer, Lyon, France; 10 Cancer Research Program, Rajiv Gandhi Centre for Biotechnology, Thiruvananthapuram, India; 11 CIBER in Epidemiology and Public Health (CIBERESP), Madrid, Spain; 12 Deutsches Krebsforschungszentrum (DKFZ), Heidelberg, Germany; Seconda Universita degli Studi di Napoli, ITALY

## Abstract

Worldwide use of formalin-fixed paraffin-embedded blocks (FFPE) is extensive in diagnosis and research. Yet, there is a lack of optimized/standardized protocols to process the blocks and verify the quality and presence of the targeted tissue. In the context of an international study on head and neck cancer (HNC)—HPV-AHEAD, a standardized protocol for optimizing the use of FFPEs in molecular epidemiology was developed and validated. First, a protocol for sectioning the FFPE was developed to prevent cross-contamination and distributed between participating centers. Before processing blocks, all sectioning centers underwent a quality control to guarantee a satisfactory training process. The first and last sections of the FFPEs were used for histopathological assessment. A consensus histopathology evaluation form was developed by an international panel of pathologists and evaluated for four indicators in a pilot analysis in order to validate it: 1) presence/type of tumor tissue, 2) identification of other tissue components that could affect the molecular diagnosis and 3) quality of the tissue. No HPV DNA was found in sections from empty FFPE generated in any histology laboratories of HPV-AHEAD consortium and all centers passed quality assurance for processing after quality control. The pilot analysis to validate the histopathology form included 355 HNC cases. The form was filled by six pathologists and each case was randomly assigned to two of them. Most samples (86%) were considered satisfactory. Presence of >50% of invasive carcinoma was observed in all sections of 66% of cases. Substantial necrosis (>50%) was present in <2% of samples. The concordance for the indicators targeted to validate the histopathology form was very high (kappa > 0.85) between first and last sections and fair to high between pathologists (kappa/pabak 0.21–0.72). The protocol allowed to correctly process without signs of contamination all FFPE of the study. The histopathology evaluation of the cases assured the presence of the targeted tissue, identified the presence of other tissues that could disturb the molecular diagnosis and allowed the assessment of tissue quality.

## Introduction

In the last decade an enormous number of molecular biology techniques have been developed, allowing for analysis of a wide spectrum of biomarkers in human specimens. Importantly, this high throughput technology permits the design of retrospective studies that are based on the use of archived material, such as formalin-fixed paraffin-embedded (FFPE) tissue samples. The conduction of worldwide multicenter studies using FFPE tissue samples has become a common practice in molecular epidemiology [1–6]. Yet, there is a lack of optimized and standardized protocols on how to process the archived FFPE tissue samples. In the case of molecular epidemiological studies, FFPE tissue blocks are processed by a broad spectrum of different procedures such as extraction of nucleic acids, immunohistochemistry (IHC) or *in situ* hybridization (ISH) analyses. For molecular studies, it is critical to avoid sample cross-contamination during the processing (e.g. sectioning) of the FFPE tissue blocks and to obtain the highest quality of the specimens in order to perform all laboratory assays. The validation of the original histopathological diagnosis is essential to ensure that the study specimens correspond to the pathology targeted by the study and that there is sufficient representation.

Studies aiming to estimate the attributable fractions (AFs) of infections-related cancers in different geographical areas are nowadays frequent. Relevant examples are the studies on mucosal high-risk (HR) human papillomaviruses (HPV) that are associated with cervical cancer and a proportion of other ano-genital cancers and oropharyngeal cancers [1–6]. In such studies, where highly sensitive assays are being used to assure the existence of invasive tumor in the paraffin curl to be tested for HPV DNA and/or mRNA, cross contamination is particularly a concern to avoid false positive results. It is also crucial to address how to avoid false negative results. This is especially relevant in head and neck cancer (HNC) studies and other tumor locations in which biopsies are usually small and the probabilities of missing relevant tissue are high. Ensuring the presence of malignant cells in the curl is important to avoid running the test on premalignant cells often seen in adjacent infiltrating tissue.

We have recently conducted a HNC case study that involved many centers in Europe and India, i.e. “Role of human papillomavirus infection and other co-factors in the aetiology of head and neck cancer in Europe and India” (HPV-AHEAD). The HPV-AHEAD consortium comprised nine partners from six European countries (Belgium, France, Germany, Greece, Italy and Spain) and one partner from India [7,8]. The main goal of the study was to perform a comprehensive analysis on a large number of HNC cases to provide important insights on the aetiology of HNC and further clarify the role of HPV infection in the disease.

Due to the multi-centric nature of the study, it was essential to develop several standardized protocols to efficiently achieve the planned goal. These protocols were to be performed simultaneously in different centres in Europe and India and included processing of human specimens for the several laboratory assays and the histopathologic review of the cases. In order to validate the standardization of the protocols, two main objectives were established. The first one was to assure that there had not been contamination when sectioning the blocks at the different centers. The second one was to confirm that the histopathology evaluation form developed by an international panel of pathologists could: 1) assure the presence of the tumor tissue, 2) identify other tissues that could invalidate the diagnosis of interest (e.g. normal or pre-neoplastic tissue) and 3) validly assess the quality of the tissue. Here, we describe all protocols and procedures for the processing of the archived FFPE tissue specimens that have been developed in the context of the HPV-AHEAD consortium and that can be adopted by other multicentric studies. We also report the results of a pilot study on the achievement of the objectives to validate it.

## Material and methods

### Protocol for sectioning of the cases

**Fig 1:**
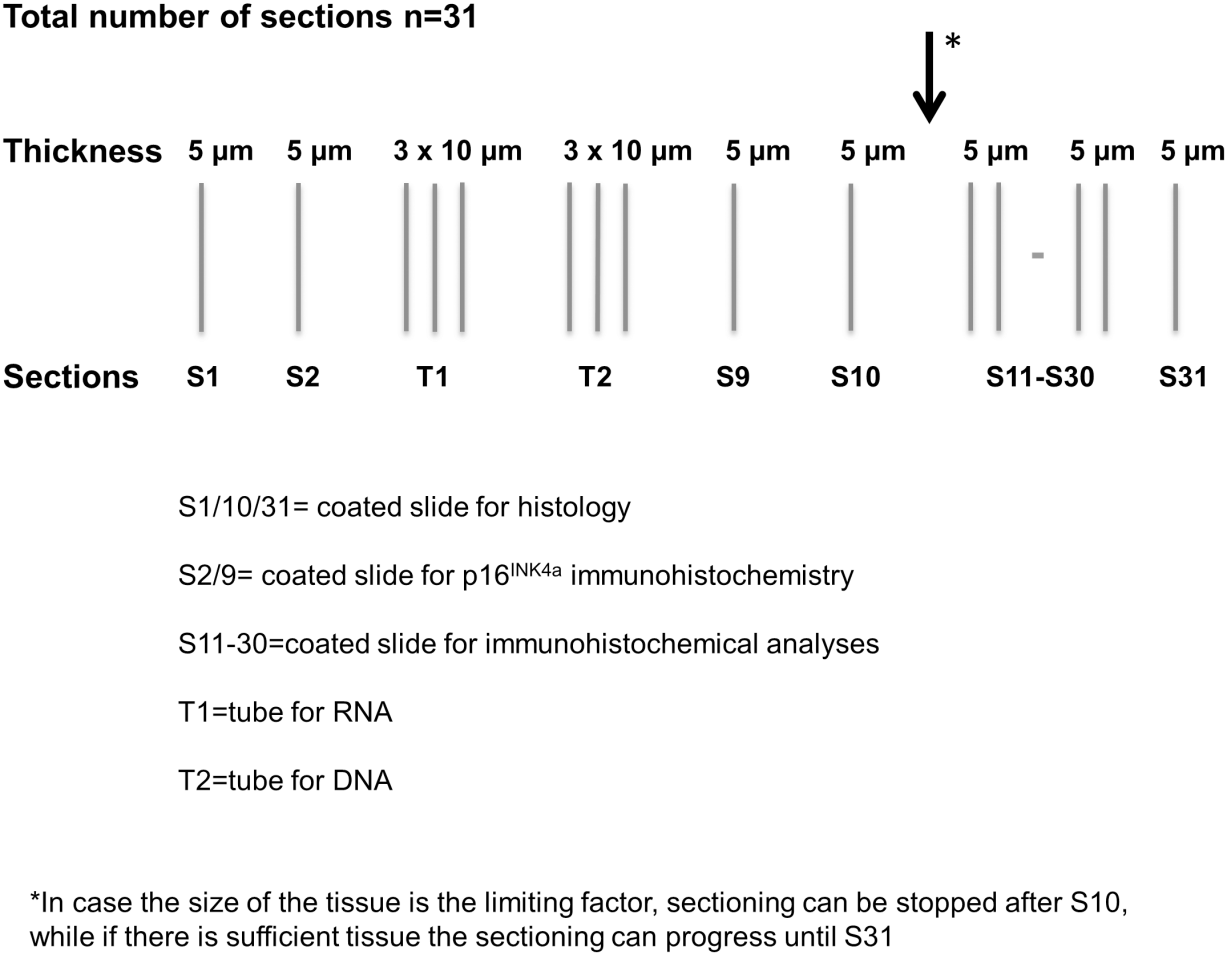
Schematic representation of the sectioning procedures.

**Fig 2:**
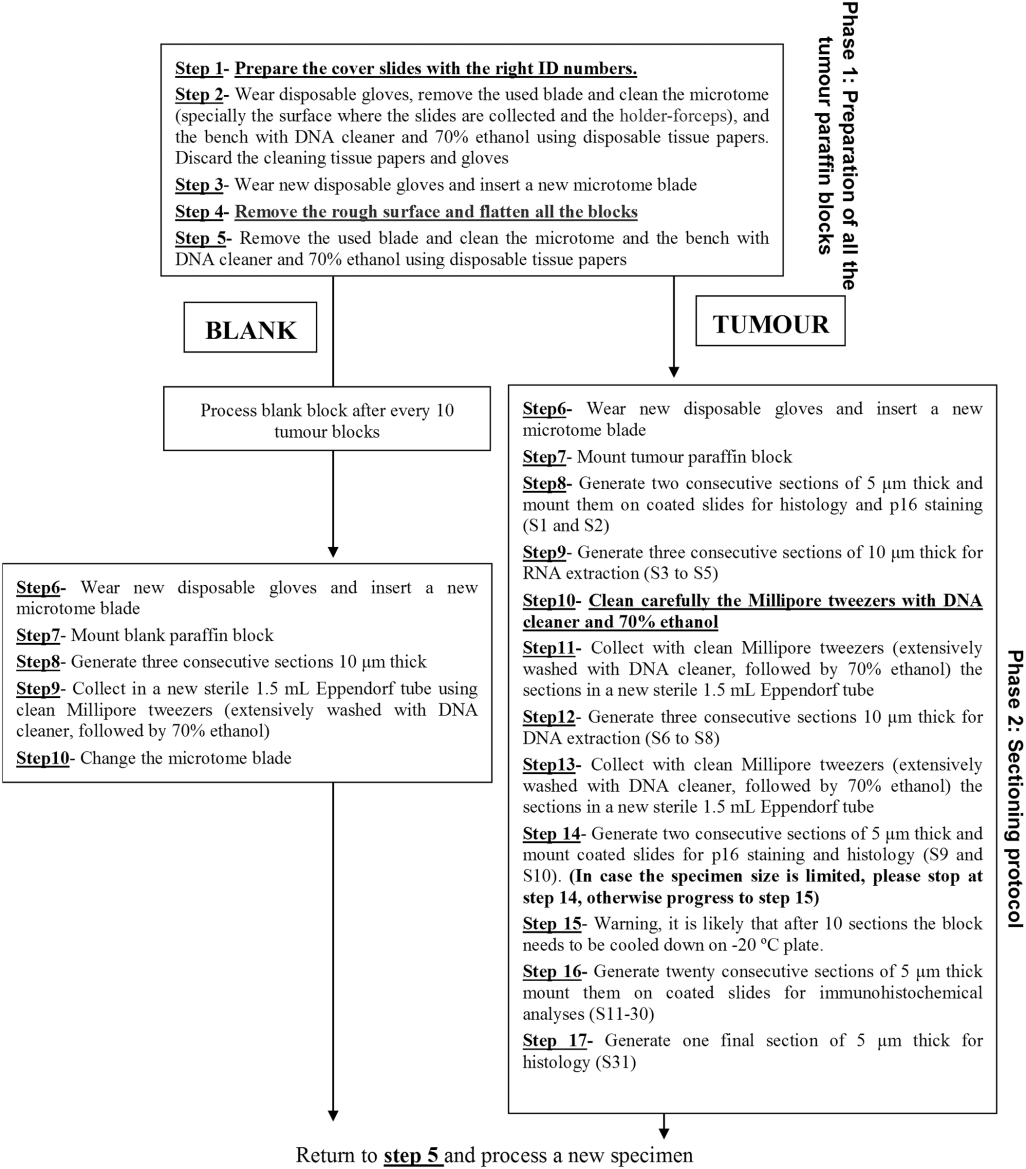
Detailed description of the sectioning procedures.

One of the major goals of the HPV-AHEAD study was to collect approximately 8000 FFPE HNCs in Europe and India and to determine the possible presence of HPV and cellular gene transcripts expression by different laboratory assays. Due to the fact that several collecting centers could not deliver the FFPE blocks to central lab of the project at the International Agency for Cancer Research (IARC), we developed a protocol for FFPE sectioning that was distributed to the collecting centers. The protocol describes the generation of several sections for laboratory assays and histological analyses, as schematically shown in Fig 1 and with more detail in Fig 2 where steps S1-S17 are described.

Only specimens fixed in neutral buffered formalin were included in the study. Specimens fixed in Bouin were not considered suitable for the study and thus excluded since the acidic environment of this fixative is a major cause for DNA degradation and makes extraction very difficult [9]. The total number of sections to be done in each block was established at 31. When the size of the tissue was the limiting factor, sectioning could be stopped after S10 (indicated by the arrows in Fig 1); otherwise the sectioning could progress until S31. The sections S1, S10 and S31 were used for histopathological assessment after haematoxylin and eosin staining. The use of the first and last section for confirmative histology was the most important aspect of sectioning, as it would guarantee that all analyses of the in-between sections were performed on the targeted cancer tissue. The second (S2) and semi-last (S9) sections were intended for p16^INK4a^ immunohistochemistry (IHC) analysis; The slides to be used for viral DNA and RNA were optimally chosen as those in between the slides for histology and p16INK4a staining: the S3 to S5 sections for HPV mRNA analysis; the S6 to S8 sections for detection of and genotyping HPV DNA. If there was sufficient material, additional slides (S11-S30) were generated for additional IHC and real-time RT-PCR analyses with other cellular proteins. The procedure was well validated in the context of the HPV-AHEAD study, and resulted in the ideal use of FFPE tissue blocks without extensive manipulation.

Due to the different nature of the routine work performed in histology laboratories, it was essential to train each collecting center in preventing cross-contamination among the different FFPE blocks during their processing. Before starting to process the FFPE blocks, each histology laboratory received a test panel comprised of 25 alternate HPV-positive and HPV-negative FFPE blocks. To avoid the use of human material and to have unlimited source, the test panel included FFPE skin tissue of wild-type or HPV 16 E6/E7 transgenic mice. Sections were generated in the histology laboratories blinded to the HPV result and shipped to IARC for HPV genotyping. Only after performing the quality test for the generation of sections with acceptable results, each collecting center was allowed to start processing FFPE HNC tissue blocks.

The protocol also described several procedures to minimize and monitor possible cross-contamination among the different FFPE (see S1 Fig). For instance, we included empty or blank paraffin blocks that were processed together with cancer specimens. Subsequently, sections from human tissue and empty paraffin were analyzed blinded to the DNA content.

### Pathology review

The HPV-AHEAD study implied to retrieve cases from archives of European and Indian hospitals. Although all the HNC tissues were already subjected to histological diagnosis, they were re-analysed in the context of the HPV-AHEAD study. A panel of six pathologists with a regional balance was created. The panel established the general criteria for a standardized histological review (S2 Fig) and generated an online pathology evaluation form (S3 Fig). Importantly, the slides of the FFPE were digitalized with an Leica SCN400 digital scanner to 20x magnification in the central lab at IARC blind to all pathologists and a database containing all digital images of the tissue sections was generated to enable all members of the panel (i) to simultaneously access the digital images and (ii) to work online from any location in the world.

#### Pilot study on the pathology form

From the 14 items included in the histopathology evaluation form, four were selected to be explored between the first and last sections and between pathologists’ reviews in a pilot study of 355 cases in order to validate the form by assuring the presence of the targeted tissue, identifying other tissues that could contaminate the results and validly assessing the quality of the tissue. Those were: quality of tissue (satisfactory, unsatisfactory, sub-optimal), percentage of invasive carcinoma (0%, <10%, 10–50%, 50–90%, > 90%), percentage of tumor necrosis (0%, <10%, 10–50%, >50%) and presence of normal epithelium (absent, present). Indeed, optically normal mucosa may carry HPV and could contaminate or give false information regarding HPV types. For example, when multiple HPV types are detected in a PCR from a piece of tissue with tumor and normal or displastic epithelium, by means of laser-microdissection it is possible to demonstrate that tumor harbours one HPV type, and benign mucosa other [10]. The specimens were distributed over six pathologists so to guarantee that all HNC were analyzed by at least two pathologists, who used the consensus histopathology evaluation form.

### Statistical analyses of the pathology review

Data were analyzed with STATA version 13.1. The main diagnoses of the pathologists were presented as frequencies to the selected items stratified by section (first or last) and pathologist. The differences of diagnoses between type of tissue (biopsy or surgical specimen) were explored by Pearson’s chi-square test or Fisher’s exact test when appropriate. Statistical significance for all the analyses was set at the 0.05 level. Concordance percent and kappa statistics were calculated to evaluate the agreement intra-pathologist (i.e. between reading of first and last sections done by the same pathologist) and inter-pathologist (i.e. between reading of first section of pathologist 1 and 2). The Kappa statistic characterization was established as following: <0: No agreement; 0–0.20: slight agreement; 0.21–0.40: fair agreement; 0.41–0.60: moderate agreement; 0.61–0.80: substantial agreement; 0.81–1.0: almost perfect agreement. Prevalence adjusted bias adjusted kappa (Pabak) statistic was explored when Kappa was not valid (i.e. when there was an unbalanced distribution of the cell counts in the 2x2 tables. Pabak statistic may be explored when high percentages of concordances (>80%) but low kappas are observed). McNemar test p-value was also calculated to evaluate the distribution among the discordant cases.

Ethical clearance for the investigations reported in this study was obtained from the Institutional Ethical Committees of MGIMS, Sevagram, India and IARC, Lyon, France. Study implied the use of archival material only, and it did not envisage any contact with the patients. Adequate measures to ensure data protection, confidentiality, patients’ privacy and anonymization were taken into account. No informed consent was available due to the retrospective design of the study and the large proportion of deceased and untraceable patients.

## Results

### Sectioning and processing of the blocks

In Europe, the HPV-AHEAD consortium retrieved approximatively 5000 FFPE HNC from 44 hospital archives, while in India 3000 FFPE HNC were retrieved in three rural and three urban hospitals. They were distributed for processing to six different histology laboratories from France, Belgium, Italy, Spain and India. As first step, each histology laboratory received the test panel of HPV-positive and HPV-negative FFPE tumor blocks containing skin tissue of wild-type or HPV 16 E6/E7 transgenic mice together with the detailed protocol for sectioning and for preventing cross-contamination of the specimens (see S1 Fig). Sections were all sent to the central lab of the study at IARC for HPV testing. Sections generated in four histology laboratories did not show any sign of cross-contamination (i.e. the results of tested cases perfectly matched the panel), while two centers required further instructions and support to implement the strict cutting procedure in their routine histology laboratories. In particular, the centers were asked to wash the sectioning platform and microtome blade extensively with DNA cleaner and 70% ethanol and wear a fresh clean lab coat and face mask (see S1 Fig). The second attempt resulted in the generation of high quality sections.

After completion of the training phase, each center processed the FFPE blocks according to the HPV-AHEAD protocols and sections were sent to the different laboratories for additional analysis. The sectioning of all FFPE tissue blocks was completed in the following histology laboratories: (i) European Institute of Oncology (n = 1075); (ii) University of Antwerp (n = 1084); (iii) Catalan Institute of Oncology (n = 1364); (iv) IARC (n = 1524) and (v) RGCB (n = 389). HPV genotyping was performed blinded to the anatomical site by a very sensitive Luminex-based assay at IARC [11, 12]. Although the assay is not considered the gold-standard, it has been validated in comparative studies in which specimens were HPV genotyped with other broadly used assays, i.e. GP5+/6+ [12, 13] and linear array (Roche Diagnostics) [14] and the results clearly showed that the Luminex assay is more sensitive than the assays mentioned above. This feature is crucial, in particular when poor quality DNA is analysed, as fragmented DNA extracted from FFPE tissue blocks [15], as in this study. Moreover, the assay, in addition to 21 HR HPV types, detects the beta-globin gene, which is used as tool to evaluate the DNA quality. In the majority of the centers we did not detect any beta-globin positivity in the empty paraffin blocks that were processed after every tenth FFPE HNC specimens. However, in two centers (IARC and RGCB), weak beta-globin positivity was detected in less than 5/147 and 2/39 of the empty paraffin blocks, respectively. Those centers were then informed and asked to follow strictly the protocol and process a smaller number of specimens per day. Most importantly, no HPV DNA was found in sections from empty paraffin blocks generated in any histology laboratories of HPV-AHEAD consortium. In addition, the generated sections were successfully used for the other laboratory analyses, including IHC staining, detection of HR HPV RNA and histology.

### Pilot study on the pathology review of the cases

The analysis of the 355 HNC histopathology evaluation forms showed that most of samples (88.7%) were biopsies and 11.0% surgical pieces (for one sample the type of tissue was not recorded). For 75.5% of the samples, S1&S31 could be evaluated (i. e. there was enough tissue for complete sectioning). This percentage was higher in surgical pieces than biopsies (94.8% vs 73.0%, p = 0.004). Most biopsies (86%) were small with a median size of 7 mm. However, on average 86% of samples were considered satisfactory in both first and last sections irrespective of being a biopsy or a surgical piece. More than 60% of the samples showed >50% of invasive carcinoma in the section. Substantial necrosis (>50%) was present in less than 2% of the samples, and thus should not affect the quality of molecular analyses. Normal epithelial components were present in approximately 61% of the samples. This could be checked to justify discordance between biomarkers (e.g. cases positive for HPV DNA but negative for p16^INK4a^). Inter-pathologist (i.e. between diagnoses of pathologists 1 and 2) differences in the diagnoses targeted to validate the form between biopsies and surgical pieces were explored. However, the results were inconsistent (data not shown), probably due to the small number of surgical pieces.

The results of the intra-pathologist (i.e. between diagnoses of first and last slides) and inter-pathologist concordance analyses of this pilot are shown in Table 1. Concordance intra-pathologist was almost perfect for the diagnoses targeted to validate the form. Concordance for diagnosis of tissue quality, percentage of invasive carcinoma, percentage of tumoral necrosis and presence of normal epithelium was very high (percentage of concordance above 92% and kappa statistics above 0.85, Table 1).

**Table 1:** Concordance of selected diagnoses of 355 HNC cases included in the pathology review by slide and pathologist.

Histological characteristics	First reading (Pathologist 1)				Review (Pathologist 2)				Inter-pathologist concordance*			
	First slide		Last slide		First slide		Last slide		% Concordance	Kappa (p-value)	Pabak	McNemar test p-value
	n	%	n	%	n	%	n	%				
**Quality of tissue**									86.1	0.34 (<0.001)	0.72	0.86
Satisfactory	308	86.8	304	85.6	308	86.8	307	86.5				
Unsatisfactory	25	7.0	34	9.6	25	7.0	30	8.5				
Sub-optimal quality	15	4.2	10	2.8	20	5.6	17	4.8				
% Intra-pathologist concordance	96.8				96.6							
Kappa (p-value)	0.85 (<0.001)				0.86 (<0.001)							
McNemar test p-value	0.03				0.31							
**Percentage of invasive carcinoma**									36.5	0.11 (<0.001)	NA	0.11
0%	12	3.4	15	4.2	11	3.1	17	4.8				
<10%	32	9.0	27	7.6	31	8.3	30	8.5				
10–50%	64	18.0	61	17.2	64	18.0	61	17.2				
50–90%	118	33.2	115	32.4	157	44.2	152	42.8				
> 90%	97	27.3	95	26.8	67	18.9	57	16.1				
% Intra-pathologist concordance	91.9				93.3							
Kappa (p-value)	0.89 (<0.001)				0.90 (<0.001)							
McNemar test p-value	0.53				0.07							
**Percentage of tumoral necrosis**									60.7	0.25 (<0.001)	0.21	0.18
0%	189	53.2	185	52.1	216	60.9	208	58.6				
<10%	86	24.2	79	22.3	74	20.9	68	19.2				
10–50%	27	7.6	29	8.2	29	8.2	30	8.5				
>50%	7	2.0	8	2.3	5	1.4	5	1.4				
% Intra-pathologist concordance	96.6				97.4							
Kappa (p-value)	0.94 (<0.001)				0.95 (<0.001)							
McNemar test p-value	0.27				0.34							
**Normal epithelium**									73.6	0.44 (<0.001)	0.47	0.06
Absent	138	38.9	137	38.6	123	34.7	120	33.8				
Present	207	58.3	202	56.9	228	64.2	224	63.1				
% Intra-pathologist concordance	96.7				96.2							
Kappa (p-value)	0.93 (<0.001)				0.92 (<0.001)							
McNemar test p-value	0.37				0.78							

NA: Not applicable. For some variables the cases do not sum 355 because of missing values or slides not evaluated.

*Calculated by comparing the first slides’ diagnoses

The concordance inter-pathologist was substantial for the diagnosis of tissue quality (satisfactory, unsatisfactory, sub-optimal): Percentage of concordance of 86%, kappa 0.34 (pabak 0.72). In contrast, the concordance in the diagnosis of percentage of invasive carcinoma was low: 37%, kappa 0.11; when grouping the diagnoses of percentage of invasive carcinoma (<50%, >50%) the concordance slightly improved: 67%, kappa 0.25. The concordance in diagnosis of percentage of tumoral necrosis was fair: 61%, kappa 0.25. In this case, when grouping the diagnoses (<50%, >50%) the concordance improved substantially: 98.3%, kappa 0.44 (pabak 0.97). Finally, the concordance in diagnosis of presence of normal epithelium was moderate: 74%, kappa 0.44.

## Discussion

To the best of our knowledge, this is the first attempt to develop and report a standardized sectioning and histopathology review protocol for optimizing the use of HNC paraffin blocks in multi-centric molecular epidemiological studies. Moreover, this protocol could also be extended for other cancer sites in which cross contamination and precise diagnosis are crucial. An equivalent histopathology protocol for breast cancer in situ has been described [16]. Another study identified aspects that could affect next generation sequencing regarding the tissue quality in solid tumors [17]. Also, a study on non-small cell lung cancer samples demonstrated considerable variation in tumor nuclei percentage between pathologists and consequently proposed a new system for automated tumor annotation and percentage tumor nuclei measurement [18]. We developed a thorough protocol for sectioning in order to avoid cross-contamination among the different blocks to be performed simultaneously in different centres of different countries. All the centers had good results in the quality control, confirming the validity and wide applicability of our protocol that it could be easily established in different histology laboratories from low and high income countries. With the increase of technological developments that allow running a wide range of biomarkers in a single sample, the need of optimized and standardized protocols such as the one herein presented is warranted. One could argue that tissue microarrays may be a more efficient way to optimize the paraffin blocks [19]. However, its applicability in international multicentric studies is very limited since there are many legal constraints to irreversibly manipulate the blocks and moreover, the risk of contamination is high.

The assessment of the histopathology evaluation form developed by an international panel of pathologists confirmed its validity to assure the presence of the targeted tissue, identify other tissues that could contaminate the diagnosis and assess the quality of the tissue. The concordance between first and last slides was very high, confirming no loss of targeted tissue throughout the different sections. Most cases were considered satisfactory regardless of the type of sample (i.e. biopsy or surgical piece). The concordance between pathologists’ diagnoses was fair to high and improved when grouping the categories of the items to more general diagnoses. Macrodisssection was not among the required procedures in our protocol and thus, the objective of assessing percentage of invasive carcinoma did not need to be as accurate as in other studies [18]. Thus, the obtained estimates of the amount of tumor tissue to confirm that tumoral DNA was not diluted with other tissues (normal, inflammatory, dysplastic, etc) were fair enough for pursuing our goals. Moreover, a database containing all digital images of the tissue sections was generated to enable all members of the panel to simultaneously access the digital images and to work online from any location in the world.

It is expected that such standardized and validated sectioning and histopathology protocol can be adapted to any multi-centric study with similar aims as the ones observed in the HPV-AHEAD study. Less stringent protocols using this scheme as backbone could also be applied to other molecular studies, including high-throughput and next generation genomic studies. The versatility of the histopathology evaluation form, which was developed specifically for the study and not for clinical practice, allows for a wide range approach studies. The protocol as presented can be easily performed in any location in the world. The herein developed procedure for simultaneous on-line revision of the samples facilitates and enhances the establishment of international panel of experts of any pathology.

In an era of increasing technological developments that allow running a wide range of biomarkers in a single sample, as well as of increase of international collaborative projects to tackle and provide important insights of a wide range of diseases, we believe that the sectioning and histopathology protocol that we have developed and validated in the context of the HPV AHEAD study may be of great interest and applicability.

## Supporting information

S1 Fig Summary of precautions to be employed during sectioning paraffin blocks for HPV analysis. (TIF)

S2 Fig Revised pathology evaluation form. (TIF)

S3 Fig Screenshot of the website pathology evaluation form. (TIF)
