# Supplementary figures and images for: Development and validation of a protocol for optimizing the use of paraffin blocks in molecular epidemiological studies: The example from the HPV-AHEAD study
Source: PLoS One. 2017 Oct 16;12(10):e0184520. doi: 10.1371/journal.pone.0184520 (PMC5642890; doi:10.1371/journal.pone.0184520)

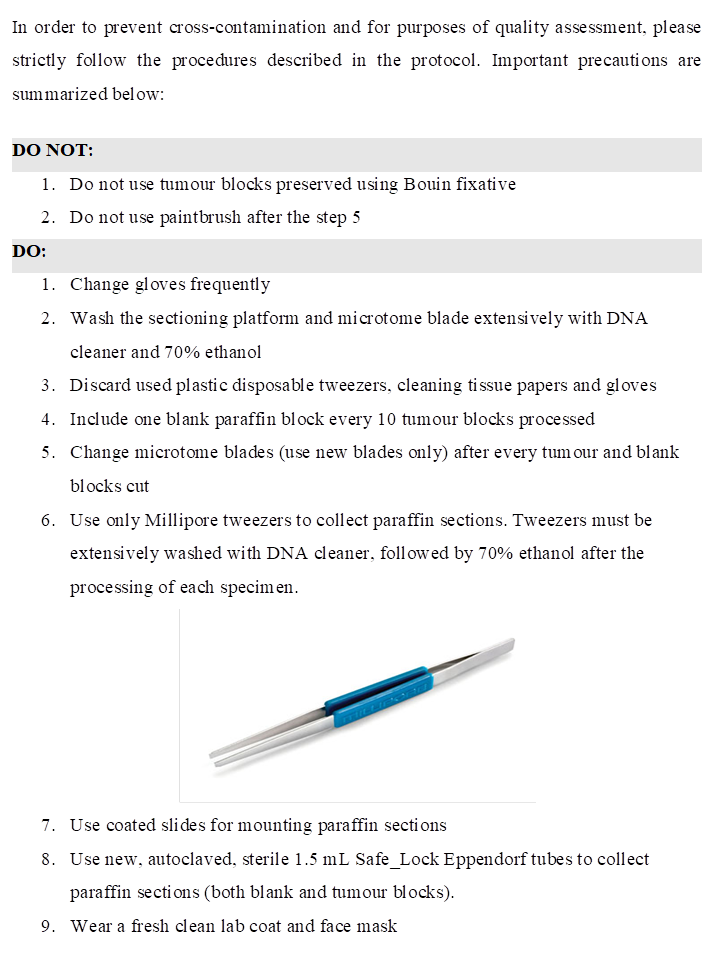

Supplement: S1 Fig — (TIF) [file pone.0184520.s001.tif]

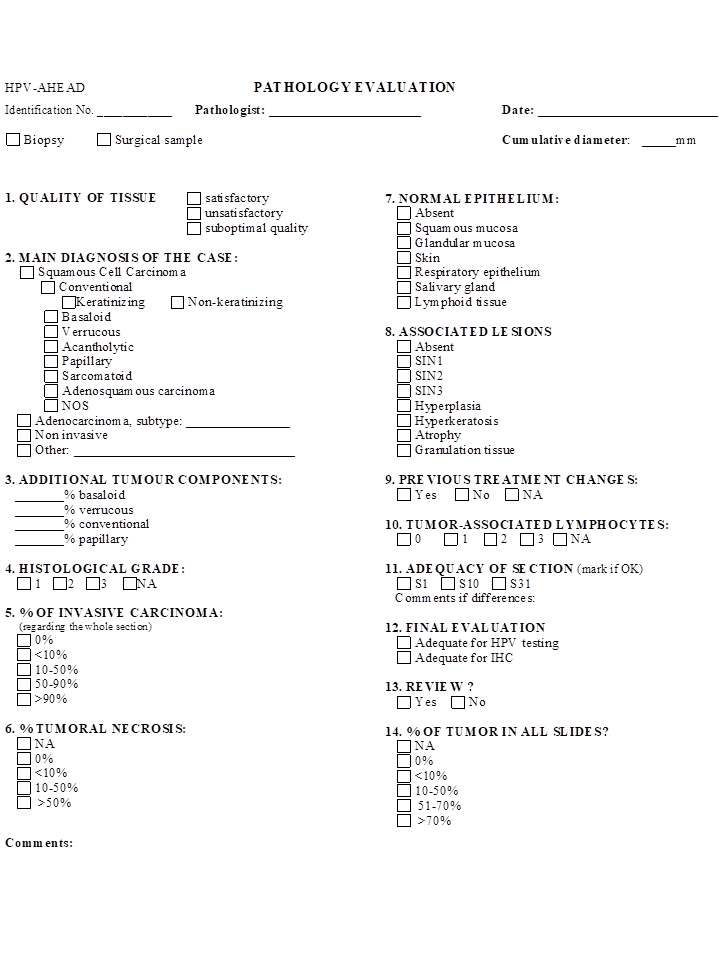

Supplement: S2 Fig — (TIF) [file pone.0184520.s002.tif]

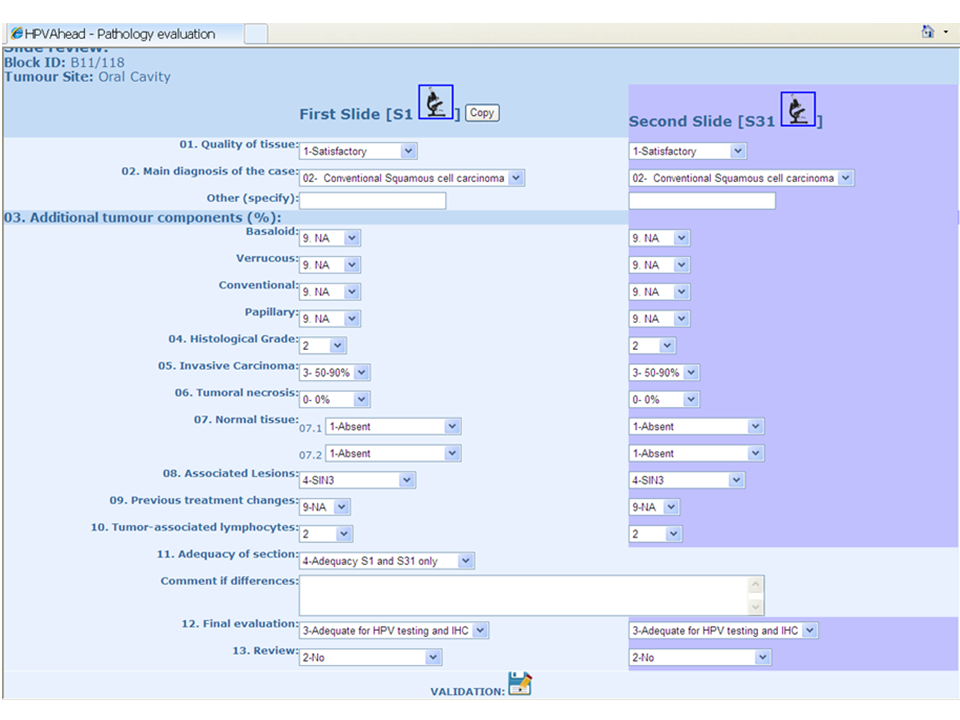

Supplement: S3 Fig — (TIF) [file pone.0184520.s003.tif]
